# Shoot the Message, Not the Messenger—Combating Pathogenic Virulence in Plants by Inhibiting Quorum Sensing Mediated Signaling Molecules

**DOI:** 10.3389/fpls.2017.00556

**Published:** 2017-04-12

**Authors:** Ganesh Alagarasan, Kumar S. Aswathy, Munusamy Madhaiyan

**Affiliations:** ^1^Department of Plant Molecular Biology and Biotechnology, Indira Gandhi Krishi Vishwavidyalaya Raipur, India; ^2^Department of Agricultural Microbiology, Tamilnadu Agricultural University Coimbatore, India; ^3^Biomaterials and Biocatalyst, Temasek Lifesciences Laboratory, National University of Singapore Singapore, Singapore

**Keywords:** biotization, Quorum sensing, pesticide poisoning, endophytes, Quorum quenching

## Abstract

Immunity, virulence, biofilm formation, and survival in the host environment are regulated by the versatile nature of density dependent microbial cell signaling, also called quorum sensing (QS). The QS molecules can associate with host plant tissues and, at times, cause a change in its gene expression at the downstream level through inter-kingdom cross talking. Progress in controlling QS through fungicide/bactericide in pathogenic microscopic organisms has lead to a rise of antibiotic resistance pathogens. Here, we review the application of selective quorum quenching (QQ) endophytes to control phytopathogens that are shared by most, if not all, terrestrial plant species as well as aquatic plants. Allowing the plants to posses endophytic colonies through biotization will be an additional and a sustainable encompassing methodology resulting in attenuated virulence rather than killing the pathogens. Furthermore, the introduced endophytes could serve as a potential biofertilizer and bioprotection agent, which in turn increases the PAMP- triggered immunity and hormonal systemic acquired resistance (SAR) in plants through SA-JA-ET signaling systems. This paper discusses major challenges imposed by QS and QQ application in biotechnology.

## Introduction

The use of synthetic broad-spectrum fungicides/bactericides in plant disease management results in imbalances within the microbial community and the continuous evolution of multiple bactericide-resistant strains. Microbes with the ability to produce quorum quenching enzymes, which can degrade the wide-spread quorum sensing signals from pathogenic microbes, could be employed in development of sustainable methods of suppressing virulence expression and abolishing bacterial infection. Quorum quenching enzymes produced by endophytes have a more limited selection pressure for microbial survival than biocide treatments (Cirou et al., 2012). Endophytic bacterial growth in plants aids disease control and promotes plant growth (Nowak, 1998; Senthilkumar et al., 2008; Jie et al., 2009; Cirou et al., 2012). Quorum quenching endophytic microbial inocula, primarily bacteria, can be used as propagating priming agents for co-culturing with plant tissues under *in vitro* conditions. This practice is an emerging trend in biotechnological approaches that harbors unprecedented potential for efficient control over virulent pathogens.

Microbial cell signaling is a precise mechanism involving many factors in play. It is now clear that the transmission of signals from synthesis to sensing depends and varies among organisms and host environments. Virulence-contributing factors like extrapolysaccharide (EPS), degradative exoenzymes, horizontal gene transfer (HGT), (Seitz and Blokesch, 2013), and effectors' secretion are controlled in a cell density-dependent manner in several plant pathogens (Helman and Chernin, 2015). Quorum sensing control of these determinants prevents the early production of factors like EPS, which could interfere with other important processes that govern invasion, such as adhesion (Koutsoudis et al., 2006).

Prokaryotes and eukaryotes have both coexisted and survived for billions of years. During this time period, both were exposed to various signaling molecules produced by each other (Shiner et al., 2005; Hughes and Sperandio, 2008). Although the existence of interkingdom signaling is predictable, the specificity of the ligands and the functions that are regulated are unique to each signaling circuit (Rampioni et al., 2014). Decoding the language taking place between plants and bacteria will be a major challenge for future research due to the numerous and different associations and/or interactions taking place in nature. This article gives a summary of advances in quorum quenching microbial research with a focus on plant-microbe interactions and the impact of QS signal molecules on the cells and tissues of plants.

## Major gene family involved in bacterial quorum sensing

QS-based microbial cell signaling aids pathogenicity of the most of pathogens (Chevrot et al., 2006; Frederix and Downie, 2011) but also helps in plant growth promotion interaction with plants (Brencic et al., 2005; Soto et al., 2006; Downie, 2010). Acyl homoserine lactone (AHL)-based quorum sensing is present in pathogens as well as many beneficial microbes, such as *Methylobacterium* (Poonguzhali et al., 2007a,b). Many Gram-negative plant-associated bacterial pathogens have been reported to regulate their virulence by AHL-based QS (Helman and Chernin, 2015). These plant pathogenic bacteria fall within a large number of species among the *Pseudomonas* and *Ralstonia* (Mansfield et al., 2012) that cause severe damage to crops.

A major bacterial intercellular signaling system in Gram-negative bacteria is LuxI/R quorum sensing based on the production (via the LuxI-family proteins) and detection (via the LuxR-family proteins) of AHL signaling molecules. Schaefer et al. (2013) screened many genomes in the Proteobacteria taxon for the presence of LuxI and LuxR homologs. Though LuxI and LuxR homolog pairs exist in Alpha-, Beta-, and Gammaproteobacteria, many isolates having LuxI/LuxR were not found to produce AHLs. LuxR proteins that have the same modular structure as LuxRs but are devoid of a cognate LuxI AHL synthase are called solos. LuxR solos have been shown to be responsible to respond to exogenous AHLs and AHLs produced by neighboring cells (Ferluga and Venturi, 2009; Gonzalez and Venturi, 2013). The LuxR-like solo protein OryR transcriptional regulator of *Xanthomonas oryzae* pv. oryzae interacts with an unknown rice signal molecule (RSM) to activate plant virulence genes (Ferluga and Venturi, 2009). Such LuxR-like solos function as messengers of both interspecies and interkingdom signaling (Gonzalez and Venturi, 2013).

## Interkingdom signaling

Plants seem to respond differently to AHL-biomolecules, which points to the existance of different receptors or signaling cascades (Götz-Rösch et al., 2015). However, until now, no specific AHL-receptor has been identified in plants. Perez-Montano et al. (2013) reported the existence of AHL-mimic QS molecules in diverse *Oryza sativa* (rice) and *Phaseolus vulgaris* (bean) plant samples. These bimolecular analogs bind to signal receptors of bacteria, but they fail to do the signaling activity of AHLs, resulting in confusing bacterial populations. A thorough analysis using biosensors carrying the lactonase enzyme showed that rice and bean seed extracts contain biomolecules that lack lactones' typical ring of AHLs. Although Götz-Rösch et al. (2015) believe that the bacterial AHL molecule might positively influence plant growth, evidence is lacking. However, plant-influenced gene expression in the rice endophyte *Burkholderia kururiensis* M130 was reported (Coutinho et al., 2015). Captivatingly, these AHL-mimicking molecules specifically alter the QS-regulated biofilm formation of two plant microbes, *Sinorhizobium fredii* and *Pantoea ananatis*, suggesting that plants can enhance or inhibit bacterial QS systems depending on the bacterial strain (Perez-Montano et al., 2013). Further studies would contribute to a better understanding of plant-bacteria relationships at the molecular level.

## The interplay between quorum-sensing molecules and phytohormones

The problem of increasing pathogens resistance to antibiotics/pesticides has prompted the search for phytometabolites with anti-QS activities (Nazzaro et al., 2013; Tan et al., 2013). However, plants have the capacity to produce secondary metabolites in smaller amounts. The considerable amount of natural anti-microbial molecule production in plants is achieved through various methods like the suspension hairy root culture and concentrations of such produced compounds were found to be sufficient for virulence suppression (Ahmad et al., 2014). Similarly, a monoterpenoid phenol carvacol demonstrated QS inhibition in bacteria, which limited biofilm formation and/or chitinase production (Borges et al., 2013; Kerekes et al., 2013; Burt et al., 2014). Phytohormones change plant-microbe interactions by orchestrating host immune responses and modulating microbial virulence traits (Xu et al., 2015). AHLs have evolved to act as interkingdom signals; many plants have been shown to respond to AHLs, which influence and alter plant gene expression (Schuhegger et al., 2006; Ortiz-Castro et al., 2008; Von Rad et al., 2008; Schikora et al., 2011; Schenk and Schikora, 2015; Schikora, 2016). These AHLs promotes plant growth in part by causing a shift in the hormonal balance between indole acetic acid and cytokinin. Long-chain AHLs that are unsubstituted at position C3 have been implicated in the modulation of root development and/or root hair formation; however, the exact mechanisms involved in recognition of microbial and/or synthetic AHLs by plant receptor proteins needs to be functionally validated. AHLs with long lipid chains that are substituted at position C3 with either a ketone or a hydroxyl have been implicated in the induction of resistance against microbial pathogens. Plants have also evolved the ability to affect bacterial AHL-QS systems given that they produce low molecular weight compounds that interfere by acting as agonists or antagonists (Adonizio et al., 2006; Degrassi et al., 2007).

## Methods for increasing the survival rate of plants during pathogenic attack

### Self-defense mechanisms in plants

Innate immunity in plants is triggered by PAMPs (pathogen-associated molecular patterns) in response to microbial infection. PAMPs are common in pathogens, non-pathogens, and saprophytes (Jeworutzki et al., 2010; Thomma et al., 2011; De Freitas and Stadnik, 2012; Vidhyasekaran, 2014). Bacterial PAMPs [e.g., certain proteins in bacterial structures and flagella, lipopolysaccharide components, muropeptides, and sugar backbone structures in peptidoglycans, the elf18 epitope in elongation factor Tu (EF-Tu), the CSP22 cold-shock protein, the Ax21 sulfated protein, rhamnolipids, superoxide dismutase (SOD), bacterial DNA, and NEP1-like proteins]; fungal PAMPs [e.g., chitooligosaccharides, ergosterol, the EIX protein, cerebrosides, and NEP1-like proteins]; and oomycete PAMPs [e.g., PEP-13, elicitins, cell wall glucans, the cell wall glycoprotein CBEL with CBD motifs, and NEP1-like proteins] are non-self-response signaling molecules recognized by plant pattern-recognition receptors (PRRs; Nicaise et al., 2009; Tsuda and Katagiri, 2010). Most PRRs are receptor-like kinase (RLK) proteins with a receptor and a signaling domain in the same molecule. In response to PAMPs, PAMP-triggered immunity (PTI) is activated (Bigeard et al., 2015), except when pathogens deliver effector proteins that interfere with PTI signaling to the host plasma membrane. In turn, plants use unique resistance (R) proteins to sense the presence of these effectors in microbes, which triggers effector-triggered immunity (ETI) (Bigeard et al., 2015).

Other intracellular signaling pathways contribute to plant immunity as well. The calcium ion, a regulator of gene expression in plants, is an intracellular second messenger involved in various defense signaling pathways in plants (Galon et al., 2010). Calcium molecules are mainly recognized by calcium sensors, which transduce calcium-mediated signals into downstream events (Hashimoto et al., 2012; Wang et al., 2012). Guanosine triphosphate (GTP)-binding proteins (G-proteins) act as molecular switches in the signal transduction system (Yalowsky et al., 2010; Zhang et al., 2012). Reactive oxygen species (ROS) and nitric oxide (NO) are highly diffusible second messengers that act in cellular signal transduction pathways. Also mitogen-activated protein kinases (MAPKs) form important signaling cascades, which act as a second line of defense in concert with PAMP. MAPKs modules are major pathways downstream of sensors/receptors that transduce extracellular stimuli into intracellular responses in plants (Hettenhausen et al., 2012). In addition to PAMP and MAPK, plant hormones such as salicylic acid (Mukherjee et al., 2010; Dempsey et al., 2011), jasmonate (Sheard et al., 2010; Bertoni, 2012), ethylene (Nambeesan et al., 2012), abscisic acid (Yazawa et al., 2012), auxin (Fu and Wang, 2011), cytokinin (Choi et al., 2011), gibberellins (Qin et al., 2013), and brassinosteroids (Vleesschauwer et al., 2012) play an important role in defense signaling against various pathogens (Vidhyasekaran, 2014). Although microbes employ various defense mechanisms to counter the pathogen attack, these mechanisms fail when pathogens reach a maximum population size.

### Inhibiting AHL production

An effective defense strategy is to block cell-signaling pathways in pathogens to arrest their growth in the host environment. The LuxI and AinS families of Acyl-HSL synthase produce AHL signals using SAM and Acyl-Acp as substrates (Gilson et al., 1995; Parsek et al., 1999). SAM analogs and the SAM biosynthesis inhibitor cycloleucine can inhibit AHL production (Hanzelka and Greenberg, 1996; Parsek et al., 1999). Mutations in AHL biosynthesis genes have direct effect on signal synthesis and biofilm formation. Mutant *P. aeruginosa* lasI, failing to synthesize 3OXOC12-HSL, forms a flat, unstructured biofilm in a flow cell. Likewise, many other mutants (e.g., *B. cenocepacia* K56-2 cepI, J2315 cepI, and cciI) are defective when grown in biofilms (Huber et al., 2001; Hentzer and Givskov, 2003; Tomlin et al., 2005; McCarthy et al., 2010; Udine et al., 2013).

### Inhibiting Rgg pheromone receptors to arrest QS in gram-positive bacteria

Rgg-class proteins are transcriptional regulators on the cytoplasmic membrane that act as receptors for intracellular signaling peptides. They are found in low-G+C-content, Gram-positive bacteria (*Firmicutes*) communication mediated by peptide molecules (Chang et al., 2011). Domain architecture prediction in Rgg proteins has revealed domains similar to that of another family of regulators (RNPP: Rap, NprR, PlcR, and PrgX) that is responsible for peptide interaction (Cook and Federle, 2014). Recently, cyclosporine, a cyclic peptide compound, was found to curb the activity of Rgg peptide receptors (Parashar et al., 2015). Though they remain crucial windows into peptide-based signaling in Gram-positive bacteria, the synthesis, and processing of Rgg peptides have not been well-studied. Further attention to this field may result in the discovery of new, effective quenching molecules against these peptide receptors.

### Autoinducer-2 inhibitors: anti-quorum-sensing molecules

Autoinducer AI-2 is a common signaling molecule used in both intra- and interspecies communication. It is a furanosyl borate diester molecule, unique due to the presence of boron in its structure (Pereira et al., 2013). Quenching of the AI-2 molecule helps in broad-spectrum control of pathogens (Zhu and Li, 2012; Guo et al., 2013; Pereira et al., 2013), as this molecule acts as a universal language for bacterial interaction. Quenching can be accomplished either by inhibiting signal biosynthesis or by inhibiting signal detection by microbes (LaSarre and Federle, 2013). Different bacterial species sense the AI-2 molecule in different forms, so a single inhibitor cannot be used widely; however, targeting the LuxS protein—which is wholly responsible for the synthesis of AI-2—results in defective signaling and is more effective in controlling a wide range of pathogens. 5'-methylthioadenosine nucleosidase (MTAN) inhibitors play dual roles as quorum quenchers in AI-2 and AHL biosynthesis (LaSarre and Federle, 2013). Halogenated furanoes, such as brominated furanoes derived from the red alga *Delisea pulchra*, have a direct role in inhibiting AI-2 quorum sensing (Lennen, 2007). Increasing the concentration of *in vitro*-produced AI-2 has a negative impact on biofilm density (Auger et al., 2006). Several diol-containing compounds (including pyrogallol), boronic acids, and sulfones have been shown to be potent antagonists of AI-2-LuxP binding (Lowery et al., 2005, 2009; Frezza et al., 2006, 2007; Ni et al., 2008, 2009; Peng et al., 2009).

### Biotization in plants and future prospects

Cross-kingdom interaction leads to specific adjustments and physiological adaptations in colonized eukaryotes (Hartmann and Schikora, 2012). The process by which non-native microbes are introduced into a plant environment is termed *biotization*. Biotization in the rhizosphere region helps plants obtain more transition metals through siderophore production, which in turn increases plant immunity against phytopathogens. Other evidence also shows that plants with hyper-accumulation of metal have increased resistance to pathogens (Fones and Preston, 2013). In order to successfully colonize a host, microbes undergo several modifications. *R. solanacearum* appears to alter its membrane architecture in complex ways during adaptation to life in the xylem (Poussier et al., 2003; Brown and Allen, 2004). Thus, culturing of beneficial microbes with plant cells in *in vitro* conditions can be used for endophytic colonization in plants (Senthilkumar et al., 2008). In plants, endophytes have an advantage over epiphytes, in that they are protected from external growth-limiting factors such as temperature, UV radiation, and osmotic potentials.

**Figure 1:**
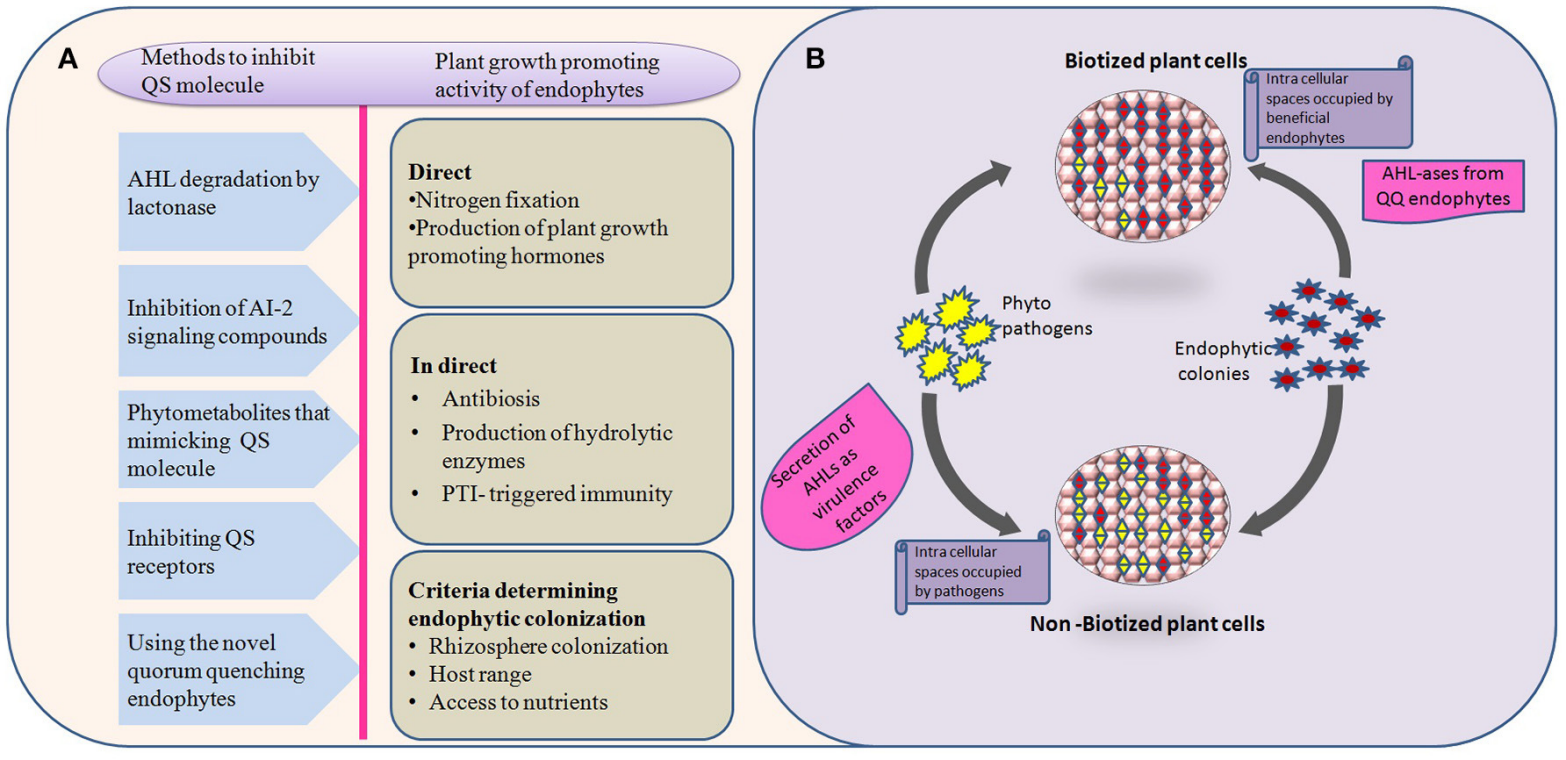
**Behavior and interactions of quorum quenching endophytes with pathogens in both biotized and non-biotized plants. (A)** The flow chart explains various methods employed by natural, and/or synthetic quorum quenching molecules to disrupt the bacterial cell signaling (left side) and potential benefits to the plants by introducing non-native endophytes (right side). **(B)** Interactions of pathogens with biotized plant tissue. The phenomenon explains the virulence activity of pathogens, and antagonistic effect of endophytes over pathogens in pre-biotized and non-biotized plants.

Various quorum quenching endophytes that have been identified in plants with AHL-ase activity are presented in Table 1. The main advantage of the artificial introduction of quorum quenching bacteria into plants is that introduced bacteria will occupy most of the intercellular spaces without leaving space for later-invading pathogenic bacteria, as shown in Figure 1. Also, biotization prevents soil bacteria entering into plant tissue (Kung and Almeida, 2014). Though a few pathogenic bacteria enter into the plant system, they remain as avirulent strain due to quorum quenching activity. Virulence-expressing factors are suppressed by AHL-degrading enzymes (Figure 1). Absence of endophytes in the tissues of culture-propagated plants may be related to increased disease severity, an idea supported by Jie et al. (2009).

**Table 1:** **Quorum quenching endophytes that have been identified in plants with experimental evidence**.

**Phylum**	**Host plant**	**Endophytic organisms**	**Disrupts QS of pathogens**	**References**
Firmicutes	*Cannabis sativa* L.	*Bacillus licheniformis, Bacillus megaterium, Bacillus pumilus, Brevibacillus borstelensis, Bacillus subtilis*	*C. violaceum*	Kusari et al., 2014
	*Ventilago madraspatana*	*Bacillus cereus VT96*	*Pseudomonas aeruginosa* PAO1	Rajesh and Rai, 2016
	*N. tabacum*	*Bacillus* sp., *Lysinibacillus* sp.	Tobacco pathogens	Ma et al., 2013
	^**^	*Paenibacillus, Staphylococcus*	*Xanthomonas campestris* pv. *campestis*	Newman et al., 2008
	*Pterocarpus santalinus*	*Bacillus firmus* PT18	*Pseudomonas aeruginosa* PAO1	Rajesh and Rai, 2014
	^**^	*Staphylococcus* sp.	*S. marcescens, P. aeruginosa,V. harveyi, C. subtsugae*	Chu et al., 2013
*Proteobacteria*	*Pterocarpus santalinus*	*Enterobacter asburiae* PT39	*Pseudomonas aeruginosa* PAO1	Rajesh and Rai, 2014
	*N. tabacum*	*Acinetobacter* sp., *Serratia* sp.	Tobacco pathogens	Ma et al., 2013
	*Oryza sativa*	Burkholderia sp. KJ006–engineered with Aii gene of *Bacillus thurungiensis*	*Burkholderia glumae,Erwinia carotovorum*	Cho et al., 2007
	^**^	*Pseudomonas(Mutants of carAB)*	*Xanthomonas campestris* pv. *Campestris*	Newman et al., 2008
Actinobacteria	^**^	*Streptomyces* sp.	*Pectobacterium carotovorum* ssp. Carotovorum	Chankhamhaengdecha et al., 2013
	^**^	*Microbacterium*	*Xanthomonas campestris* pv.campestris	Newman et al., 2008
	Colonization of plant surfaces	*Arthrobacter, Mycobacterium Nocardioides, Rhodococcus*, and *Streptomyces*	*N*-oxododecanoyl-L-homoserine lactone, N-hexanoyl-L-homoserine lactone	Polkade et al., 2016
Basidiomycota	*Saccharum officinarum*	*Rhodotorula* yeasts	*Chromobacterium violaceum* CV026	Bertini et al., 2014
Ascomycota	*Ventilago madraspatana*	*Fusarium graminearum* and Lasidiplodia sp.	*Chromobacterium violaceum* CV026	Rajesh and Rai, 2013
	Marine endophytic fungi	*Sarocladium (LAEE06), Fusarium (LAEE13), Epicoccum (LAEE14), and Khuskia (LAEE21)*.	*Chromobacterium violaceum CVO26*	Martin-Rodriguez et al., 2014
	Plant rhizosphere	Ascomycota and Basidiomycota	C6HSL and 3OC6HSL	Uroz and Heinonsalo, 2008

** *Indicates non-native plant endophytes but experimentally proved to have quorum quenching activity*.

### Barriers in successful biotization

So far there are no universal QQ bacterai to be used for all plants. Also, biotization has been shown to be limited by the absence of AHL-based quorum sensing in Gram-positive bacteria, instead inhibition of Rgg pheromone receptors could be employed (Cook and Federle, 2014). Research has shown that there is a chance that bacteria can evolve resistance to QS-disruption-related control methods (Defoirdt et al., 2010). Hence, a deep understanding of plant-microbe interactions in both biotized and non-biotized plants should be the goal of future research. The challenges of *in vitro* biotization are summarized in Figure 1.

### Endophytes as microbial fertilizers

Biotization helps at various physiological and developmental stages in plants. It enhances induced biotic and abiotic stress resistance (Badosa and Montesinos, 2008; Lugtenberg and Kamilova, 2009; Senthilkumar et al., 2011). Endophytes improve plants' health mainly through siderophore production, thereby enabling biological nitrogen fixation (Ngamau et al., 2012), phosphate solubilization (Andrade et al., 2014), and effective transport of iron (Fe) and zinc (Zn) from the rhizosphere region by ZIP transporters (Krithika and Balachandar, 2016).

## Conclusion

The quorum quenching mechanism can serve as a potential target for developing new antimicrobials to overcome microbial pathogenesis. Quorum quenching endophytes will attenuate virulence factors rather than kill the microbes, a feature that should hugely reduce the selective pressures associated with bactericidal agents that have led to the rapid emergence of resistance. Before engineering the quorum sensing pathway in native endophytes, it is important to ensure the presence of naturally available beneficial mechanisms in the host environment. The key to improving plant resistance to bacterial diseases in a changing environment may lie in creating biotized plants. Pesticide poisoning has been acknowledged as a serious problem in many agricultural communities of low- and middle-income countries. Efforts to develop a systematic and a sustainable approach to prevent and manage pesticide poisoning remain inadequate. Thus, the novel possibility of exploiting the quorum quenching endophytes may serve as a sustainable tool for plant disease management.

## Author contributions

GA, KA, and MM initiated the project. All the authors have made a substantial, direct, intellectual contribution to the work, and reviewed the final version of the manuscript.

### Conflict of interest statement

The authors declare that the research was conducted in the absence of any commercial or financial relationships that could be construed as a potential conflict of interest.
