# Corrigendum: Shoot the Message, Not the Messenger—Combating Pathogenic Virulence in Plants by Inhibiting Quorum Sensing Mediated Signaling Molecules

**DOI:** 10.3389/fpls.2017.02198

**Published:** 2017-12-21

**Authors:** Ganesh Alagarasan, Kumar S. Aswathy, Munusamy Madhaiyan

**Affiliations:** ^1^Department of Plant Molecular Biology and Biotechnology, Indira Gandhi Krishi Vishwavidyalaya, Raipur, India; ^2^Department of Agricultural Microbiology, Tamilnadu Agricultural University, Coimbatore, India; ^3^Biomaterials and Biocatalyst, Temasek Lifesciences Laboratory, National University of Singapore, Singapore, Singapore

**Keywords:** biotization, Quorum sensing, pesticide poisoning, endophytes, Quorum quenching

**Munusamy Madhaiyan** was not included as an author in the published article. The authors apologize for this error and state that this does not change the scientific conclusions of the article in any way.

The original article has been updated.

## Author contributions

GA, KA, and MM initiated the project. All the authors have made a substantial, direct, intellectual contribution to the work, and reviewed the final version of the manuscript.

### Conflict of interest statement

The authors declare that the research was conducted in the absence of any commercial or financial relationships that could be construed as a potential conflict of interest.

